# Constructing and applying a multi-turn psychological support dialogue corpus based on the Helping Skills Chain-of-Thought

**DOI:** 10.3389/fpsyg.2026.1733384

**Published:** 2026-03-16

**Authors:** Lanqing Du, Yunong Li, Yujie Long, Shihong Chen

**Affiliations:** 1 School of Computer Science, Guangdong University of Foreign Studies South China Business College, Guangzhou, China; 2 School of Information Science and Technology, Guangdong University of Foreign Studies, Guangzhou, China

**Keywords:** Chinese corpus, helping skills theory, large language models, multi-turn dialogue, psychological support

## Abstract

With the increasing prominence of mental health issues, automated psychological support dialogue systems have gradually gained attention. However, existing Chinese corpora mostly remain at the level of single-turn Q&A or lack psychological counseling theoretical grounding, making it difficult to cover the progressive interactions common in psychological counseling. Meanwhile, collecting and releasing large-scale real multi-turn dialogues faces challenges related to privacy protection and high costs. To address this, this paper proposes the Helping Skills Chain-of-Thought (HCoT) method, which integrates Helping Skills Theory with Chain-of-Thought prompting. We utilized GPT-4o to rewrite CD-CN single-turn data into a Chinese multi-turn psychological support corpus, HCoT-Corpus. This corpus contains 22,341 dialogues and 211,473 strategy annotations, achieving a systematic expansion in scale, structural depth, and theoretical grounding. Analysis results indicate that HCoT-Corpus demonstrates high structural coherence and multi-strategy collaborative characteristics under the “Exploration-Comfort-Action” three-stage framework. Experimental evaluations show that, compared to baselines like SMILE, the HCoT method achieves the most balanced performance in emotional resonance, strategy application, and structural integrity. Furthermore, HCoT-Chat, fine-tuned on Qwen2.5-7B-Instruct, achieved significant advantages in both automatic metrics and cross-model evaluations. This study demonstrates the HCoT method as a promising path for constructing large-scale, theoretically grounded psychological support dialogue datasets.

## 1 Introduction

According to the World Mental Health Report published by the WHO (2022), approximately 970 million people worldwide are suffering from mental disorders, posing immense challenges to individuals, families, and society (Sun et al., 2021; Organization, 2022; Chen et al., 2025). Mental Health Support refers to providing responses with high relevance, helpfulness, and empathy to clients to assist them in coping with common psychological issues such as anxiety, stress, and depression (Sun et al., 2021). However, constrained by factors such as a shortage of counselors, social stigma, and high costs, many Seekers (help-seeker) cannot obtain timely mental health support. This structural supply–demand imbalance has led researchers to explore the development of AI-driven mental health support dialogue systems (Chen T. et al., 2024; Zhang C. et al., 2024; Xu J. et al., 2025).

Early psychological dialogue systems, such as ELIZA and Woebot, achieved rule-driven basic psychological response functions (Weizenbaum, 1966; Fitzpatrick et al., 2017). However, due to the lack of high-quality psychological dialogue corpora, their effectiveness was limited, making practical application difficult. Liu et al. (2021), based on the helping skills theory proposed by Hill (2009), crowdsourced the construction of the English multi-turn dataset ESConv. Building on the theoretical foundation of the ESC framework, Sun et al. (2021) constructed the Chinese psychological Q&A dataset PsyQA. With the rapid development of Large Language Models (LLMs), researchers have begun to explore utilizing their powerful language understanding and generation capabilities to enhance the quality and scalability of mental health support dialogues. Some studies have started using LLMs to rewrite and augment existing psychological dialogue data to expand corpus scale and improve generation quality (Chen et al., 2023; Liu et al., 2023; Qiu et al., 2024; Zhang C. et al., 2024). However, these studies generally fail to introduce psychological counseling theories; while the generated content is natural, it lacks the guidance of structured psychological counseling strategies. Other studies have combined psychological counseling theories with LLMs to generate Chinese single-turn responses with a professional psychological intervention style, but these efforts remain largely at the level of single-turn generation, lacking multi-turn structural design and dynamic control of support strategies (Na, 2024). Real counseling processes are inherently multi-turn and dynamic, relying on the continuous identification of the client’s state and strategy adjustment. To enhance the reasoning capability and explainability of LLMs, researchers introduced the Chain of Thought (CoT) method, improving logical consistency and task decomposition abilities by generating intermediate reasoning steps (Wei et al., 2022). Studies have confirmed that emotion-enhanced Chain of Thought not only optimizes the performance of psychological counseling models but also enhances model explainability—meaning researchers can better understand the model’s decision-making process—thereby improving its performance in mental health support tasks (Wang et al., 2023; Yang et al., 2023; Li et al., 2024; Zhang T. et al., 2024).

Based on these inspirations, this paper proposes a novel dialogue generation method that integrates Helping Skills Theory with the Chain-of-Thought mechanism: Helping Skills Chain-of-Thought (HCoT). To the best of our knowledge, this method pioneers the use of Chain-of-Thought to guide LLMs in simulating the progressive strategy rhythm and reasoning process characteristic of psychological counseling. Specifically, it leverages the “Exploration—Comfort—Action” three-stage structure and typical support strategies from Helping Skills Theory as explicit reasoning steps during multi-turn generation. The overarching objective is to produce multi-turn simulated supportive dialogues that possess both structural clarity and rational pacing.

**Figure 1:**
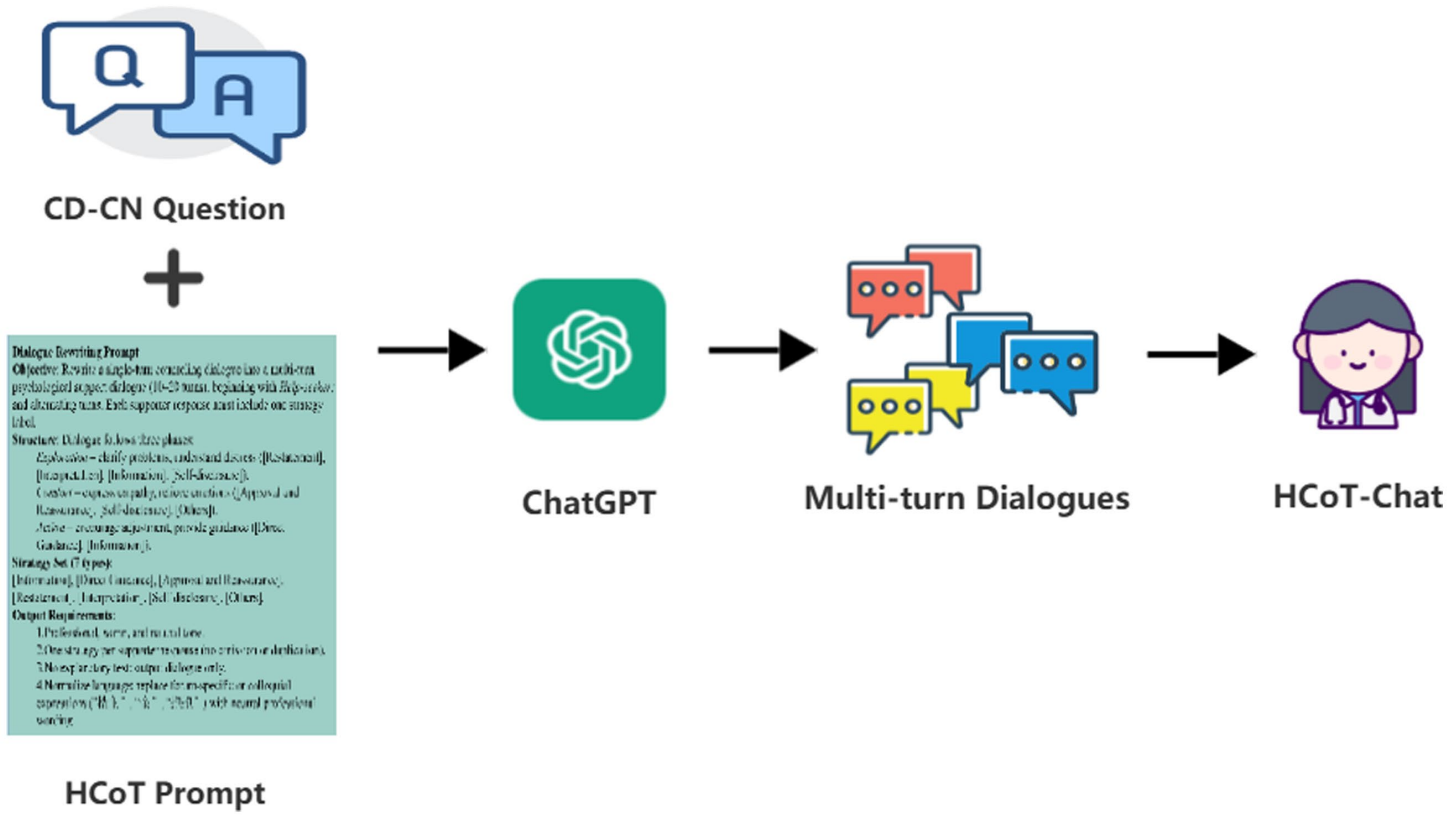
The HCoT method for generating simulated psychological counseling dialogues.

As illustrated in Figure 1, utilizing the HCoT method, we employed GPT-4o to rewrite the CD-CN dataset into a multi-turn dialogue dataset, HCoT-Corpus, annotated with specific psychological support strategies. This corpus contains 22,341 dialogues and 211,473 turns. We conducted a detailed data analysis covering lexical, semantic, and thematic features. Furthermore, results from both LLM automatic evaluation and human review consistently indicate that dialogues generated by this method demonstrate high theoretical adherence and structural integrity.

Finally, this study developed the HCoT-Chat model by fine-tuning on HCoT-Corpus and verified its performance through metrics such as BLEU, Rouge-L, METEOR, and Distinct (Papineni et al., 2002; Lin, 2004; Banerjee and Lavie, 2005; Li et al., 2016), as well as pairwise reviews. This dataset aims to provide a structured, theoretically grounded empirical corpus foundation for the fine-tuning of Large Language Models in the mental health domain.

However, developing high-quality training data for LLM-based mental health support is a multi-step, multi-disciplinary endeavor that requires defining the scope of application and strictly delimiting limitations (Xu Y. et al., 2025). This study focuses on a critical initial stage of this process: serving as a “Methodological Proof-of-Concept” (Kim et al., 2025), aimed at verifying the procedural utility of the HCoT method in generating structured, theoretically grounded dialogues. We employ low-burden evaluation methods to measure key indicators of dialogue quality, such as strategic coherence and structural adaptability (Neary et al., 2025). The goal is to determine whether HCoT is promising enough to warrant higher-burden validation (e.g., assessment by clinical experts) in the future. Through preliminary benchmarking, this work establishes HCoT as a feasible path for developing large-scale, high-quality mental health datasets and provides a necessary foundation for subsequent higher-burden research.

## 2 Related work

### 2.1 Mental health support dialogue datasets

A critical challenge currently confronting mental health support dialogue systems is the scarcity of high-quality datasets. Due to the highly sensitive nature of counseling content and the requirement for professional-level annotation, constructing such datasets is constrained by privacy concerns and prohibitive costs. In the field of Emotional Support Conversation (ESC), Liu et al. (2021), drawing on the Helping Skills Theory proposed by Hill, developed the ESC framework comprising three stages—Exploration, Comfort, and Action—along with their respective support strategies. They subsequently constructed the English multi-turn dataset ESConv, designed to train models in executing support strategies. However, as this dataset was constructed via crowdsourcing, it remains relatively small in scale (approx. 1.3 k dialogues) with high annotation costs. To mitigate these costs, recent research has pivoted toward utilizing prompt engineering for data generation. For instance, Zheng et al. (2023) leveraged seed scenarios from ESConv to guide ChatGPT in automatically generating multi-turn emotional support dialogues annotated with strategies. Nevertheless, these studies have primarily focused on emotion-alleviation tasks within English contexts and have yet to deeply explore the construction of structured mental health support datasets specifically for the Chinese context.

In the Chinese context, Sun et al. (2021) collected mental health Q&A data from online counseling websites to construct PsyQA, a dataset containing seven types of support strategy labels. However, this dataset is limited to single-turn Q&A formats and relied on costly crowdsourcing. To expand multi-turn capabilities, Qiu et al. (2024) proposed the SMILE method, employing ChatGPT to rewrite PsyQA into multi-turn dialogues. Similarly, Chen et al. (2023) combined crowdsourced single-turn corpora with emotional support prompts to guide ChatGPT in generating multi-turn empathetic dialogues. Additionally, Zhang C. et al. (2024) extracted structured information from online counseling reports and designed a reconstruction framework to restore and expand Chinese counseling dialogues. Despite these advancements in enlarging Chinese psychological corpora, most prior work has failed to systematically incorporate psychological counseling theory as a generative backbone. Consequently, existing data often lacks the structural modeling of support strategies and the stage-wise guidance mechanisms necessary to emulate the progressive, goal-directed nature of real counseling sessions.

### 2.2 Chain-of-thought prompting

Chain-of-Thought (CoT) prompting, first proposed by Wei et al., enhances the reasoning and task decomposition capabilities of Large Language Models (LLMs) by introducing intermediate reasoning steps into few-shot prompts (Wei et al., 2022). This method has been widely applied in domains such as mathematical calculation and commonsense reasoning, and has recently demonstrated significant potential in emotional support dialogue tasks. By simulating step-by-step reasoning, CoT helps models better interpret user emotional states and structure their responses. Research by Yang et al. (2023) confirmed that emotion-enhanced CoT not only optimizes the performance of psychological counseling models but also improves model explainability, allowing researchers to better understand the decision-making process behind therapeutic responses. Similarly, the CogChain method proposed by (Cao et al., 2025) simulates the cognitive process of a supporter through an “Understanding-Reasoning-Response” chain, significantly deepening the model’s comprehension of user problems. Zhang T. et al. (2024) proposed ESCoT, which introduces an “Emotion Recognition—Strategy Planning—CoT Generation” framework, validating that explicit reasoning steps can improve strategy adherence and logical consistency.

However, existing CoT research in this domain has largely concentrated on English emotional support tasks. Research involving structured modeling and reasoning synergy mechanisms for Chinese mental health dialogues remains relatively scarce. Current approaches often struggle to support the complex, long-context reasoning required for the multi-turn “Exploration-Comfort-Action” progression, highlighting the need for a framework like HCoT that integrates domain-specific theory with chain-of-thought reasoning.

## 3 Methods

Drawing on the data construction paradigm of PsyQA, we constructed the CounselDialog-CN v1.0 (CD-CN) dataset through systematic updates and expansion. As an authoritative Chinese psychological counseling corpus, PsyQA integrated content from the Q&A section of *YiXinLi*, a prominent Chinese psychological service platform. The original scale was approximately 22,000 Q&A pairs, featuring authentic, natural, and semantically complete content with a formal style, covering common themes such as interpersonal relationships, family dynamics, and emotional regulation. Given that the original data coverage is relatively dated, and considering the platform’s recent functional updates (e.g., tipping features and interaction optimization) and overall quality improvements, we collected over 20,000 of the latest Q&A samples. These samples cover user inquiries, counselor responses, and basic metadata (such as topic tags). While the platform employs a built-in content moderation mechanism to filter extreme speech and unsafe content, to further ensure ethical compliance, we employed Large Language Models (LLMs) to conduct automated text auditing. This process was used to screen and conditionally filter potentially sensitive or high-risk samples, alongside intelligent desensitization, ensuring the data is safe for model training and public research.

### 3.1 Data preprocessing

Compared to the raw “YiXinLi” corpus, real psychological counseling contexts place a greater emphasis on professionalism and standardization, necessitating the adaptation of forum-specific phrasing. Traditional approaches (Sun et al., 2021) typically employ a two-stage cleaning process involving rule-based filtering and manual review; however, such methods incur high costs in large-scale generation scenarios.

To address this, we adopted a more lightweight strategy: we directly integrated language cleaning requirements into the prompt design, instructing the model to automatically normalize phrasing during the rewriting process. For instance, forum-specific forms of address such as *“楼主”* (thread starter) or *“题主”* (questioner) are standardized to *“*你*”* (you); affectionate comforting expressions like *“抱抱”* (hugs) are removed; and colloquial social terms such as *“亲”* (dear) or *“宝”* (baby) are strictly prohibited. The overall tone is maintained as neutral, professional, and gentle.

The goal of this procedure was to reduce the manual cost of preprocessing and enforce stylistic consistency, with the aim of yielding model-generated responses that more closely resemble professional counseling-style dialogues. Please refer to Appendix A for the detailed preprocessing pipeline.

### 3.2 Task definition

Based on the psychological Q&A dataset CD-CN, this study integrates the Helping Skills Chain-of-Thought prompt template ( $P_{\text{HCoT}}$ ) and utilizes the Large Language Model GPT-4o to rewrite the CD-CN single-turn Q&A pairs into a multi-turn dialogue dataset incorporating support strategies. Specifically, for each question $q_{i}$ and its description $d_{i}$ in CD-CN, the model is guided by $P_{\text{HCoT}}$ to generate a dialogue $c_{i}$ containing multi-turn psychological support, which is formulated as:


$$c_{i}=\mathrm{GPT}-4o(q_{i},d_{i},P_{\text{HCoT}})$$

Given that the generated multi-turn dialogue $c_{i}$ has already fully integrated the contextual information of the original question and description, this paper retains only the structured dialogue content when constructing the final corpus. This approach simplifies the input structure to enhance model training efficiency. Consequently, the resulting HCoT multi-turn psychological support dialogue corpus is denoted as:


$$D_{\text{HCoT}-\text{Corpus}}=\{c_{i}\}$$

This corpus serves for the fine-tuning language models for psychological support and the evaluation of multi-turn generation performance. The overall objective is to systematically guide Large Language Models, by introducing Helping Skills Chain-of-Thought prompts, to generate psychological support dialogues characterized by stage rhythm (Exploration—Comfort—Action), strategy diversity, and contextual coherence. This aims to enhance the model’s execution capability and logical consistency regarding structured support strategies in simulated counseling scenarios.

### 3.3 Support strategies and prompt design

The strategy framework employed in this study draws upon Hill’s Helping Skills Theory (Hill, 2009), which conceptualizes the counseling process into three distinct stages: “Exploration—Comfort—Action.” Integrating the specific characteristics of the Chinese context, we adopt the six fine-grained support strategies defined in PsyQA: *[Information]*, *[Direct Guidance]*, *[Approval and Reassurance]*, *[Restatement]*, *[Interpretation]*, and *[Self-disclosure]* (see Table 1 for detailed definitions). We additionally use [Others] as a catch-all label for utterances that do not match any of the six definitions.

**Table 1:** Definitions and examples of the six support strategies.

Strategies	Definitions	Examples
Information	Supply information in the form of data, facts, opinions and resources.	*心理学中有个关于“初恋”的效应, 叫“蔡格尼克记忆效应”。* *There is a psychological effect on first love, called Zeigarnic effect.*
Direct Guidance	Provide suggestions, directives, instructions, or advice about what the help-seeker should do to change.	*如果觉得难以改变, 可以寻求靠谱的心理咨询师的帮助。* *If you find it hard to change, you can seek help from a trusted counselor.*
Approval and Reassurance	Emotional support, reassurance, encouragement and reinforcement.	*给你温暖的抱抱呀!* *Let me give you a warm hug!*
Restatement	A simple repeating or rephrasing of the content or meaning of the question, usually in a more concrete and clear way.	*您感觉自己产生了暴虐心理* *You feel like you are becoming violent.*
Interpretation	Go beyond what the help-seeker has overtly stated or recognized and give a new meaning, reason or explanation.	*我想你是很爱很爱妈妈的。* *I think you love your mom very much.*
Self-disclosure	Reveal something personal about the helper’s non-immediate experiences or feelings.	*这个问题勾起了我类似的回忆。* *This question brings back to me some similar memories.*

[Others] are used as a fallback category and is therefore not listed in this table. The functional definition of each strategy follows (Sun et al., 2021s), and we provide bilingual (Chinese–English) dialogue examples adapted to our setting. These examples serve as the basis for structural annotation and strategy identification in multi-turn dialogue generation.

**Figure 2:**
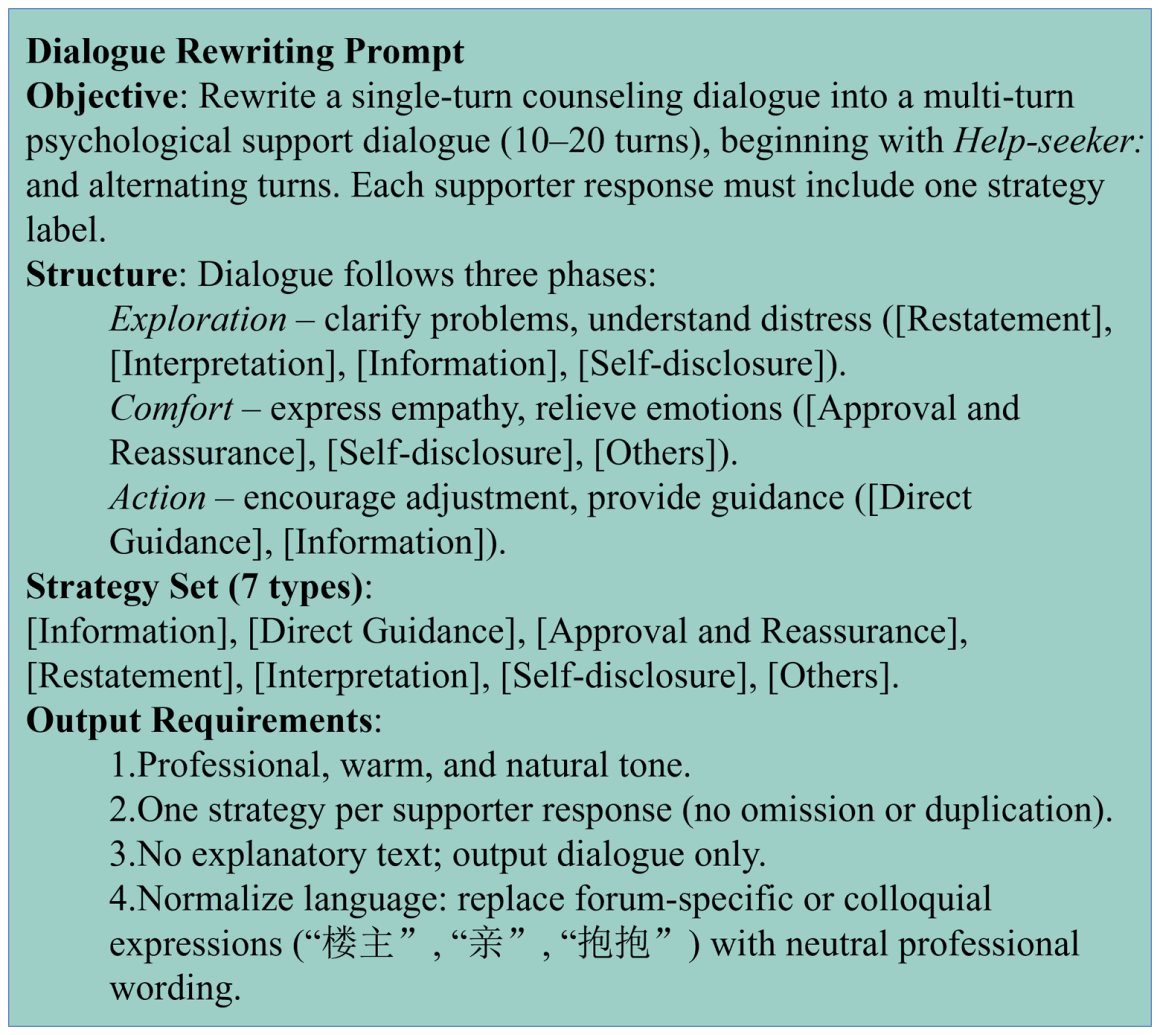
HCoT prompt used for dataset generation.

To facilitate structured generation, we designed the Helping Skills Chain-of-Thought (HCoT) prompt template (as illustrated in Figure 2). This prompt guides GPT-4o to rewrite the CD-CN single-turn Q&A pairs into multi-turn dialogues, strictly constraining the model to adhere to the three-stage evolutionary logic while explicitly embedding strategy tags within each response turn. This mechanism ensures that the generated content rigorously aligns with the norms of Helping Skills Theory in terms of semantics, pacing, and structure.

### 3.4 Data generation

In this study, GPT-4o served as the generative model to transform 22,341 single-turn psychological Q&A samples from the CD-CN dataset into multi-turn dialogues. The generation process utilized hyperparameters set to temperature = 0.7 and top_p = 0.9. The former controls randomness to ensure response coherence and prevent extreme expressions, while the latter balances diversity with semantic relevance, accommodating the gentle and empathetic requirements of psychological support dialogues. All other parameters remained at their default values.

Based on the proposed Helping Skills Chain-of-Thought (HCoT) and the aforementioned settings, we constructed the HCoT-Corpus. This dataset represents the first Chinese multi-turn mental support dialogue corpus grounded in Helping Skills Theory that systematically introduces the “Exploration—Comfort—Action” three-stage structure and support strategy annotations. It aims to provide a structured, theoretically grounded empirical corpus foundation for the fine-tuning of Large Language Models in the mental health domain.

## 4 Data analysis

### 4.1 Data filtering and cleaning

To ensure the quality of HCoT-Corpus, we filtered and cleaned the 22,341 multi-turn dialogues generated by GPT-4o, focusing on optimizing dialogue format, ending patterns, and turn counts to strictly adhere to the prompt requirements of “alternating turns, supporter-ending, and 10-20 turns.” The specific cleaning steps were as follows:

1. *Format Standardization:* Using regular expressions, we identified and removed approximately 0.5% of dialogues that violated the alternating-speaker constraint (e.g., consecutive turns by the same role), enforcing the canonical pattern: *“Seeker:”* followed by *“Supporter [Strategy]:.”*
2. *Closing Consistency:* We identified 595 dialogues (2.66%) that ended with a Seeker turn. Of these, 593 ending with simple expressions of gratitude were truncated (i.e., the final turn was removed), while 2 containing complex closing content were removed after manual review, ensuring all dialogues conclude with a supporter’s response.
3. *Turn Count Correction:* We addressed 9 dialogues with non-compliant turn counts (including 3 empty dialogues and 6 with an odd number of utterances) by regenerating them to meet the 10–20 turn constraint.

After cleaning, HCoT-Corpus comprises 22,341 dialogues, all of which fully satisfy the design specifications. This process significantly enhanced structural consistency and professionalism, laying a solid foundation for subsequent analysis and model fine-tuning.

### 4.2 Data analysis

1 *Turn count statistics*

**Figure 3:**
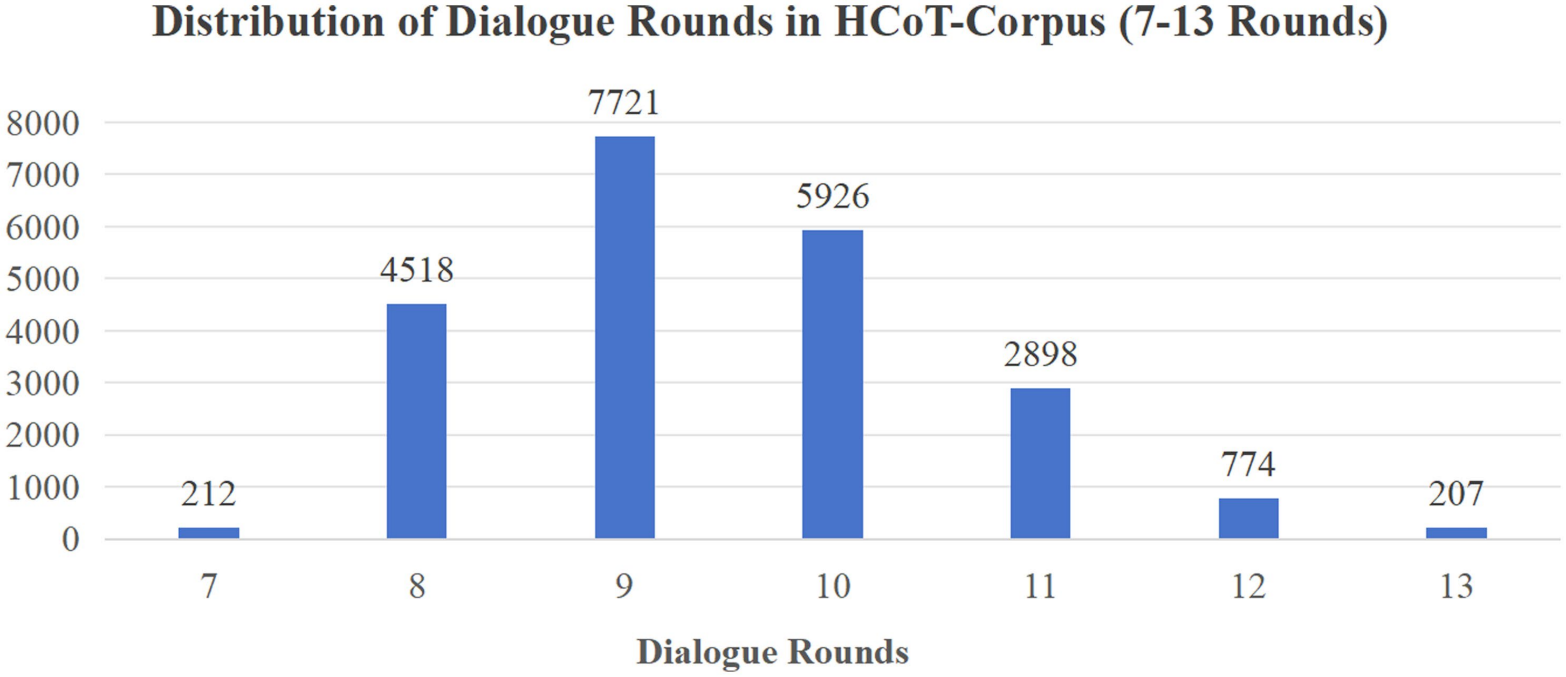
Turn count distribution in HCoT-Corpus.

After filtering and cleaning, HCoT-Corpus comprises 22,341 dialogues (approximately 430,000 utterances), with an average of 9.47 turns per dialogue (distribution shown in Figure 3). In this paper, a “turn” refers to one Seeker–Supporter exchange (i.e., two utterances).

It is worth noting that, although this average is slightly below the prompt’s preset lower limit (10 turns), this reflects the efficiency of the model in executing the HCoT framework rather than hastiness. The model is capable of fully evolving through the three stages of “Exploration—Comfort—Action” and concluding naturally within a relatively compact scope, effectively avoiding mechanical padding merely to satisfy length constraints. The high scores obtained in the “Direct Guidance” dimension in subsequent evaluations confirm that the integrity of the Action stage was not compromised by the streamlined turn count. Furthermore, compared to SMILECHAT (5.7 turns) (Qiu et al., 2024), HCoT-Corpus demonstrates a significantly increased turn count, better accommodating the depth requirements of progressive support characteristic of counseling-style interactions.

2 *Accuracy of strategy labels*

To verify the accuracy of support strategy labels in HCoT-Corpus, we randomly sampled 200 dialogues from the dataset, ensuring balanced coverage across diverse turn counts and topics. We invited three evaluators with backgrounds in psychology or related fields to participate in the assessment.

To ensure evaluation quality, all evaluators underwent systematic training on the Helping Skills theoretical framework and the strategy definitions provided in Table 1 prior to the formal evaluation. Furthermore, they passed a consistency calibration during a pre-annotation phase. Subsequently, the evaluators independently assessed the semantic consistency between the generated strategy labels and the response content.

The results indicated a label accuracy of 90.0% and a Fleiss’ Kappa coefficient of 0.85. This strong inter-rater agreement demonstrates that, following systematic training, the evaluators achieved a high degree of consensus regarding strategy definitions, ensuring high annotation reliability. The few mismatched cases were primarily attributed to boundary ambiguity between *[Others]* and *[Approval and Reassurance]*.

In summary, the scale, structural depth, and annotation quality of the dataset fully meet the requirements for fine-tuning Large Language Models in mental health contexts.

### 4.3 Analysis of strategy chain structure and distribution

#### 4.3.1 Frequency and distribution of support strategies

**Figure 4:**
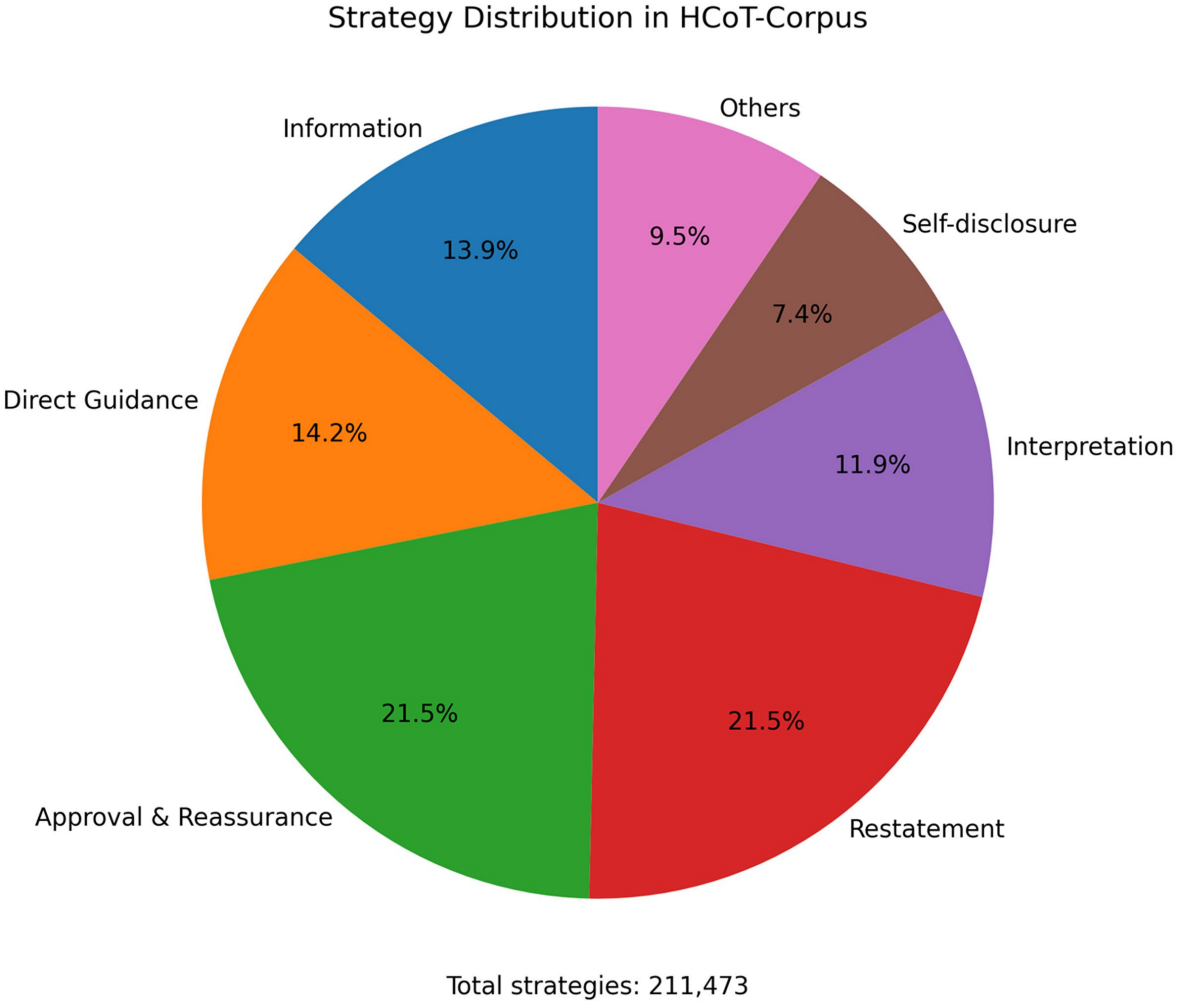
Proportional distribution of support strategies in supporter responses within HCoT-Corpus.

To investigate the deployment characteristics of support strategies within HCoT-Corpus, we analyzed the frequency and proportional distribution of the seven support strategy categories, comprising a total of 211,473 annotated strategies (see Figure 4).

Results indicate that [Restatement] emerged as the most frequently employed strategy, accounting for 24.19% of the total. This was followed by *[Approval and Reassurance]*, *[Direct Guidance]*, and *[Information]*. In contrast, the usage proportions of *[Self-disclosure]* and *[Others]* remained relatively low. This distribution aligns with the archetypal rhythm of multi-turn psychological support dialogues: initially stabilizing the supportive rapport and emotional state through restatement and reassurance, followed by advancing the topic with guidance and information provision.

This analysis not only reveals the regularity of strategy distribution in multi-turn psychological support dialogues but also provides the data foundation and theoretical underpinning for the subsequent in-depth discussion on strategy chain evolution (Section 4.3.2) and its alignment with the three-stage Helping Skills framework (Section 4.3.3).

#### 4.3.2 Strategy chain structure features analysis

To further elucidate the organizational patterns and structural characteristics of support strategies in HCoT-Corpus, we extracted all 22,341 strategy chains. Statistical analysis reveals that chain lengths are predominantly distributed between 7–13 turns (average 9.47), indicating sufficient depth of dialogue expansion.

High-frequency strategy combinations exhibit highly stable characteristics of stage evolution (see Table 2): chains typically initiate with *[Restatement]* and *[Interpretation]*, flexibly incorporate *[Self-disclosure]* and *[Approval and Reassurance]* in the intermediate phase, and predominantly conclude with *[Direct Guidance]*. This distribution precisely mirrors the theoretical rhythm of “Exploration—Comfort—Action.” Further observation of the top 10 strategy chains reveals that they collectively cover all 7 strategy types, demonstrating the model’s capacity to flexibly mobilize diverse support strategies during multi-turn interactions, rather than being confined to the repetitive use of single strategies.

**Table 2:** Statistics of high-frequency support strategy combinations in HCoT-Corpus (top 10).

Rank	Strategy chain (abbrev.)	Frequency
1	Res → Intpn→Info→A&R → Disc→Guid→Oth → A&R	310
2	Res → Intpn→Disc→Info→A&R → Guid→Oth → A&R	285
3	Res → Intpn→Info→Disc→A&R → Guid→Oth → A&R	209
4	Res → Intpn→Info→Disc→A&R → Oth → Guid→Info→A&R	208
5	Res → Intpn→Info→Disc→A&R → Guid→Oth → Info→A&R	167
6	Res → Intpn → Info → A&R → Disc → Guid → Info → A&R → Oth	154
7	Res → Intpn → Info → A&R → Disc → Guid → Oth → Info → A&R	153
8	Res → Intpn → Info → Guid → A&R → Disc → Oth → Guid → A&R	138
9	Res → Intpn → Info → A&R → Disc → Guid → A&R → Oth	135
10	Res → Intpn → Info → A&R → Disc → Oth → Guid → Info → A&R	110

These results indicate that the model successfully adheres to the phased structure defined by the HCoT prompt and achieves synergistic application of multiple strategies during dialogue progression, thereby closely approximating the dynamic process described in Helping Skills Theory.

#### 4.3.3 Analysis of three-stage structural alignment

We conducted a stagewise analysis on 450 samples randomly selected from the HCoT-Corpus dataset (covering nine common psychological themes such as interpersonal relationships, marriage, and family). Specifically, we leveraged GPT-4o to perform stage segmentation and strategy identification based on the “Exploration—Comfort—Action” framework of Helping Skills Theory, aimed at verifying whether the corpus conforms to the expected theoretical structure.

Results indicate that the vast majority of dialogues fully encompass the three-stage structure, with evolutionary logic rigorously aligning with theoretical expectations. Regarding stage sequences, the standard structure “ECA (Exploration-Comfort-Action)” was predominant, appearing in 309 instances; this was followed by variants such as “ECACA” (101 instances). This demonstrates that HCoT-Corpus generally adheres to a stable stage progression rhythm while reflecting the dynamic alternation of emotional resonance in specific samples.

**Figure 5:**
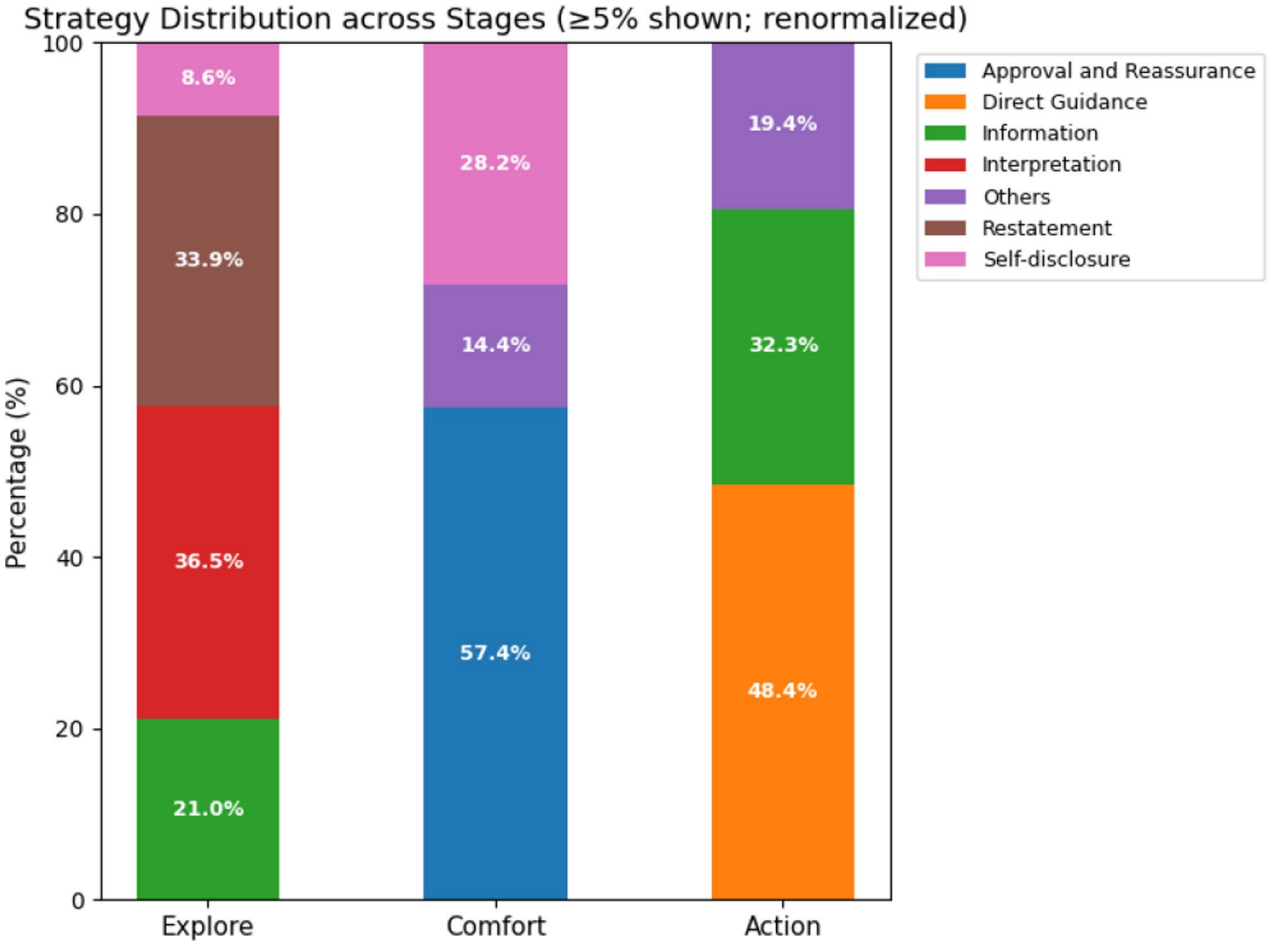
Distribution of support strategies across the three-stage structure.

Regarding strategy distribution (see Figure 5), each stage exhibits distinct characteristics corresponding to theoretical expectations:

- The Exploration stage relies heavily on *[Restatement]* and *[Interpretation]* to clarify problems;
- The Comfort stage is dominated by *[Approval and Reassurance]* to ensure emotional reception;
- The Action stage is characterized by *[Direct Guidance]*, emphasizing practical orientation.

Overall, HCoT-Corpus exhibits a clear “Exploration—Comfort—Action” rhythm, validating the procedural utility of the HCoT method in structural control and theoretical alignment. Nevertheless, a minority of samples still exhibit stiff transitions (e.g., from Exploration to Action), and the highly standardized “ECA” structure reflects the idealized characteristics of synthetic data compared to real counseling. This suggests that future model optimization should further prioritize the naturalness and flexibility of dialogues.

### 4.4 Comparative experiments

To systematically validate the procedural utility of the HCoT framework as a data generation method across its two core components—"Macro-Structure” and “Micro-Strategy”—we designed a comparative experiment. Regarding data selection, we constructed a test set comprising 450 single-turn dialogue samples based on HCoT-Corpus using stratified random sampling. This sample set uniformly covers nine common psychological counseling themes, such as interpersonal relationships, marriage, and family, ensuring the generalizability of experimental results across diverse counseling contexts.

Subsequently, we performed rewriting on the aforementioned 450 samples using the GPT-4o model. To disentangle the independent contributions of theoretical guidance at different granularities to generation quality, we established the following three experimental groups:

1. *SMILE method (no-theory baseline):* Proposed by (Qiu et al., 2024), this represents a general LLM rewriting paradigm without psychological counseling theoretical guidance, utilizing concise prompts to guide text expansion. Serving as a baseline, it aims to establish the performance benchmark of models relying solely on general linguistic capabilities in the absence of domain knowledge constraints, thereby validating the necessity of introducing professional theories.
2. *3Stage method (macro-structure only):* As a structured variant of the HCoT framework, this method retains the “Exploration—Comfort—Action” macro-structure from Helping Skills Theory but ablates the explicit guidance of micro-level support strategies (e.g., *Restatement*). This control group is designed to quantify the independent gain of specific counseling strategies in enhancing dialogue professionalism and empathetic depth by controlling variables.
3. *HCoT method (full method):* This represents the complete framework proposed in this paper, integrating both the three-stage macro-structure and the micro-strategy chain-of-thought. This group aims to validate the comprehensive utility of the “Structural Planning + Strategic Guidance” synergistic mechanism in simulated counseling scenarios.

Crucially, to eliminate the potential “Label Halo Effect” caused by explicit strategy tags and ensure a fair comparison across these groups, we implemented a strict “Tag Removal” protocol during the evaluation phase. Specifically, explicit strategy markers (e.g., [Restatement]) were stripped from the HCoT-generated content to standardize them into natural dialogue formats. This ensured that all evaluations were conducted under a “blind” condition, focusing solely on the semantic quality, empathy depth, and logical coherence of the text.

Ultimately, by systematically comparing these three paradigms—General Generation (SMILE), Macro-Structure Only (3Stage), and Macro–Micro Synergy (HCoT)—this experiment aims to intuitively reveal the differences in generation quality caused by different granularities of theoretical guidance across dimensions such as emotional resonance, strategic richness, and logical coherence. This process not only confirms the structural compliance of the HCoT framework but also critically analyzes the independent contributions of “Macro-Structure” and “Micro-Strategy” in dialogue generation, providing empirical reference for the optimization of future psychological support dialogue models.

#### 4.4.1 Experimental design

The evaluation of psychological counseling dialogues requires balancing semantic accuracy, emotional support intensity, and strategy appropriateness. To ensure objective comparison, this experiment employed GPT-5.2 as an automatic evaluator, leveraging its superior capabilities in complex semantic understanding to perform fine-grained scoring. During the experiment, the model’s temperature parameter was uniformly set to 0.3, while other parameters were kept at default values to guarantee the stability and reproducibility of the evaluation results.

This study adopts the Bench evaluation framework proposed by June (2023). This framework comprises seven core dimensions that align highly with professional helping strategies in real counseling, aiming to comprehensively measure the model’s execution capability regarding professional strategies. The definitions of each dimension are as follows:

- *Information:* Providing clear, accurate, and relevant factual information or resources.
- *Direct guidance:* Offering explicit action suggestions, instructions, or guiding the user toward behavioral change.
- *Approval and reassurance:* Providing emotional support and affirmation to enhance user security and confidence.
- *Restatement:* Accurately paraphrasing, clarifying, or confirming content expressed by the user.
- *Interpretation:* Going beyond surface information to analyze the user’s emotions, motives, or underlying meanings.
- *Self-disclosure:* Moderately revealing the supporter’s own non-immediate experiences or feelings to promote empathy and trust.
- *Gathering:* Collecting necessary details through effective questioning to drive the dialogue deeper.

During scoring, GPT-5.2 independently rated the aforementioned dimensions (on a scale of 0–10) based on preset system instructions.

#### 4.4.2 GPT-5.2 automatic evaluation

Regarding overall performance (see Table 3), the HCoT method demonstrates the most balanced and superior performance, achieving the highest Total Score (37.34). Notably, while maintaining robust scores in key dimensions such as *[Direct Guidance]* and *[Interpretation]*, HCoT established a decisive advantage in the most challenging dimension, [Self-disclosure] (3.54), vastly outperforming 3Stage (0.02) and SMILE (0.03). This reflects HCoT’s breakthrough in emotional holding and human-like interaction.

**Table 3:** GPT-5.2 automatic scoring results of three methods across seven psychological support dimensions.

Evaluation dimensions	SMILE	3Stage	HCoT
Information	3.45	3.45	**4.51**
Direct Guidance	5.60	6.01	**6.48**
Approval and Reassurance	5.95	7.12	**7.38**
Restatement	4.83	6.20	**7.08**
Interpretation	4.39	5.50	**6.08**
Self-disclosure	0.03	0.02	**3.54**
Gathering	5.89	**7.52**	2.27
Total	30.14	35.82	**37.34**

Bold values indicate the best performance/highest scores in each respective category.

In contrast, while the 3Stage method (Total = 35.82) leveraged its structural framework to achieve the highest score in *[Gathering]* (7.52), it exhibited insufficient depth due to the lack of emotional strategies (such as *[Restatement]* and *[Self-disclosure]*). SMILE demonstrated the weakest overall performance (Total = 30.14), limiting its comprehensive support capabilities. This indicates that HCoT better balances emotional support with strategic guidance.

**Figure 6:**
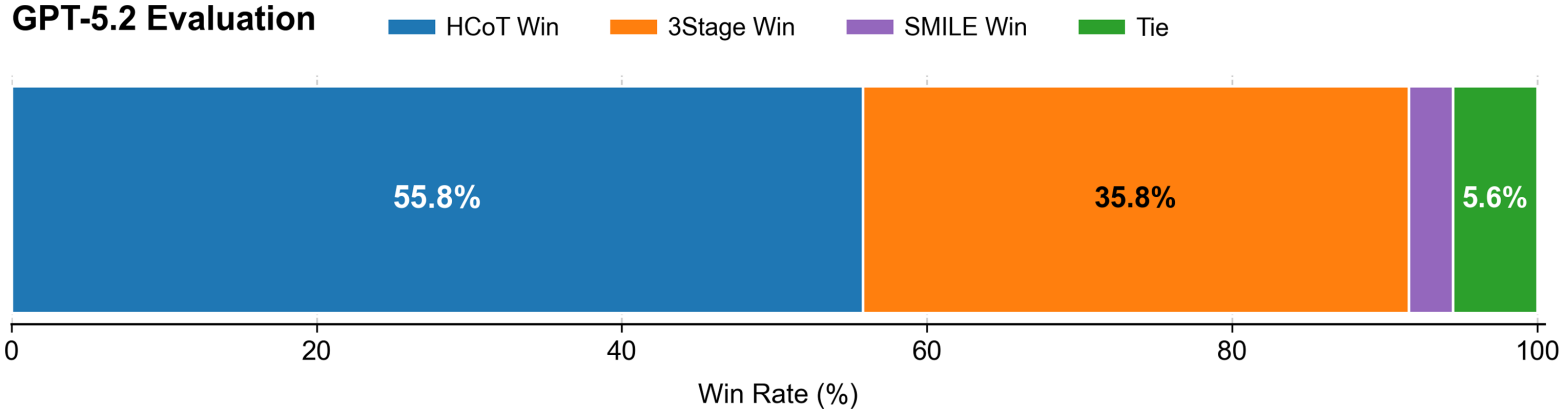
Distribution of optimal preferences for the three methods based on GPT-5.2.

Furthermore, the simultaneous “Best-of-Three” comparative evaluation corroborates these trends (as shown in Figure 6). It should be noted that the “Total Score” is the simple sum of the seven-dimension scores, whereas “Best-of-Three” is the evaluator’s final preference judgment; the two serve as complementary indicators. In the preference determination executed by GPT-5.2, the HCoT method was selected as “Best” in 55.78% (251/450) of the test samples, a proportion significantly higher than that of 3Stage (35.78%) and SMILE (2.89%). The remaining samples were labeled as ties (Tie) when the evaluator could not confidently determine a single best response and were therefore excluded from the win-rate counts of all methods. This preference distribution aligns closely with the multi-dimensional scoring results, confirming that the synergy between “theoretical structural integrity” and “micro-strategy richness” emphasized by HCoT aligns more closely with the evaluator’s definition of high-quality psychological support.

#### 4.4.3 Human evaluation

**Figure 7:**
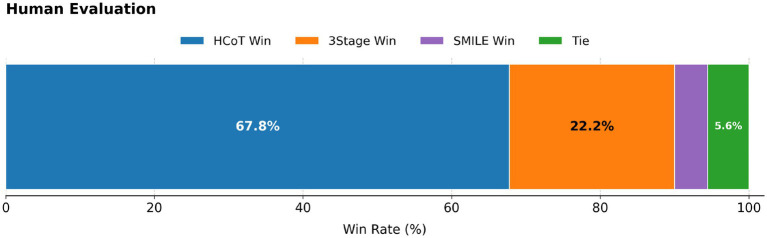
Human preference distribution of the three methods (90 dialogue groups; each group contains three responses from HCoT/3Stage/SMILE). The winner label is aggregated from the three trained raters’ best-of-three judgments via majority vote (2:1 yields a winner; 1:1:1 is labeled as a tie). Results: HCoT 67.8%, 3Stage 22.2%, SMILE 4.4%, and ties 5.6%.

To further validate the reliability and effectiveness of the automatic scoring mechanism, this study conducted a human evaluation experiment. The rating team consisted of three graduate and undergraduate students with backgrounds in psychology or computer science, consistent with the demographic profile described in Section 4.2. To ensure professional rigor, all members underwent systematic training on psychological counseling evaluation, including multiple rounds of trial scoring and expert calibration. They proceeded to the formal evaluation phase only after their weighted Cohen’s Kappa value during calibration consistently exceeded 0.7. During the formal evaluation, the raters independently scored a full set of 90 dialogue groups (totaling 270 items) randomly selected by topic. In addition, to enable sample-level preference alignment, we asked raters to provide an extra Best-of-Three preference judgment for each group (choosing among HCoT/3Stage/SMILE). The final winner label was aggregated by majority vote (2:1); cases without a majority were marked as ties (see Figure 7). The results yielded an average pairwise weighted Cohen’s Kappa of 0.78, demonstrating high inter-rater reliability and stable scoring criteria (this Kappa is computed based on the seven-dimensional 0–10 ordinal ratings, rather than the Best-of-Three preference labels).

A comparative analysis between automatic and human scoring revealed a significant “scale shift” phenomenon: GPT-5.2 employed more stringent criteria than human raters, resulting in systematically lower absolute scores for the HCoT, 3Stage, and SMILE methods. Despite the disparity in absolute values, the aggregate performance ranking remained consistent (HCoT > 3Stage > SMILE). Direct statistical tests further supported this alignment: the agreement rate between GPT-5.2 and the trained human raters on “Best Model Determination” (Top-1 Accuracy) reached 65.56%, and the overall preference ranking exhibited a stable positive correlation (Kendall’s Tau = 0.46). These results provide evidence that the AI evaluator’s preferences are broadly aligned with those of the trained human raters in distinguishing higher-quality responses (Table 4).

**Table 4:** Human scoring results of three dialogue generation methods across psychological support dimensions.

Evaluation dimensions	SMILE	3Stage	HCoT
Information	6.29	6.08	**7.11**
Direct Guidance	7.74	7.52	**8.42**
Approval and Reassurance	7.08	**7.52**	7.49
Restatement	5.82	6.47	**6.91**
Interpretation	6.66	7.09	**7.92**
Self-disclosure	2.87	2.77	**6.44**
Gathering	7.99	**8.43**	5.86
Total	44.45	45.88	**50.15**

Bold values indicate the best performance/highest scores in each respective category.

Furthermore, at the fine-grained dimensional level, both evaluations consistently attributed an overwhelming advantage to HCoT in the [Self-disclosure] dimension (Human 6.44 vs. AI 3.54). The win rate of HCoT in human evaluation (67.8%) aligns closely with the distribution trend of the automatic evaluation results (55.78%), further supporting GPT-5.2’s ability to discriminate model performance. Qualitative feedback also indicated that evaluators generally agreed that the generated responses across all groups effectively eliminated forum-style characteristics of the original data, demonstrating high professionalism and standardization, thereby supporting the effectiveness of the pre-processing strategies outlined in Section 3.1.

#### 4.4.4 Evaluator independence verification

Despite the robust performance of GPT-5.2, relying on a single evaluation perspective introduces the potential risk of Self-preference Bias. To mitigate this, we introduced Grok-4.1 and Gemini-3-Pro-Preview (hereafter Gemini-3), models with distinct architectures, to conduct cross-validation.

Analysis of the fully aligned data confirmed the high robustness of the evaluation results: GPT-5.2 exhibited a significant positive correlation in scoring trends with the third-party arbiter models (GPT vs. Grok: ρ=0.56 ; GPT vs. Gemini: ρ=0.45 ; all p<0.001 ). Furthermore, to verify precise consistency at the decision-making level, we analyzed 400 decisive samples (from an original N = 450) after excluding ambiguous ties—cases without a decisive preference across the three evaluators. Results indicate that the three models achieved a 50.50% unanimous agreement rate, far exceeding the random baseline for a three-class task (~11%). Meanwhile, the pairwise Cohen’s Kappa coefficients between GPT-5.2 and the two arbiter models (Grok-4.1 / Gemini-3) were 0.38 and 0.33, respectively, indicating reasonable cross-model consistency.

On this basis, results from the arbiter models (see Table 5; detailed scoring tables and win-rate distributions for each arbiter are provided in Appendix B) show that the HCoT method consistently maintained a lead across all evaluators (HCoT > 3Stage > SMILE). Notably, HCoT received cross-architectural recognition in the [Self-disclosure] dimension, compellingly demonstrating the consistent advantage of this method in counseling-style support strategies, rather than being an artifact of single-model evaluation bias.

**Table 5:** Comparison of cross-model evaluation consistency (N = 450).

Evaluator model	HCoT win rate	Total Score ranking	Self-disclosure score
GPT-5.2	55.78%	HCoT > 3Stage > SMILE	3.54
Grok-4.1	70.22%	HCoT > 3Stage > SMILE	5.89
Gemini-3	54.67%	HCoT > 3Stage > SMILE	7.34

## 5 Dialogue system

The objective of this study is to construct a high-quality multi-turn dialogue dataset based on Helping Skills to simulate psychological counseling interactions. Assessing the quality of a dialogue dataset is non-trivial and is typically achieved indirectly through dialogue systems. Consequently, this study involves training a dialogue system and analyzing its performance.

### 5.1 Mathematical formulation

To train a dialogue system for psychological support, we first split each full dialogue d~D into multiple training sessions. Specifically, a sampled t-turn dialogue session can be represented as: $d_{t}=\{u_{1},r_{1},u_{2},r_{2},\ldots ,u_{t},r_{t}\}∼D$ .

We then train a dialogue model to predict the supporter response $r_{t}$ conditioned on the dialogue history $h_{t}=\{u_{1},r_{1},u_{2},r_{2},\ldots ,u_{t}\}$ . Our objective is to fine-tune a large language model $\pi_{0}$ on D via supervised learning, i.e., maximum likelihood estimation:


$$J_{\mathrm{SFT}}(\theta )=E_{(h_{t},r_{t})\sim D}[\log\pi_{\theta }(r_{t}|h_{t})]$$

Where $\pi_{\theta }$ is initialized from $\pi_{0}$ .

### 5.2 Data preparation

We utilized the HCoT-Corpus dataset, randomly partitioning it into a training set (90%) and a test set (10%). To align with the formatting requirements for instruction-based fine-tuning, the dialogues were segmented into multiple sessions, with each session concluding with a supporter’s final utterance.

Crucially, adhering to the “Tag Removal” protocol established in Section 4.4, we explicitly stripped strategy tags from the training data. Although the raw HCoT-Corpus contains explicit markers (e.g., *“Supporter [Information]:”*), we standardized the role format to a clean “Supporter:” for the instruction-tuning dataset. This processing ensures that the model performs natural end-to-end generation, preventing the leakage of non-natural language tags during inference, while compelling the model to implicitly learn the strategic logic and semantic patterns embedded within the text.

Furthermore, following the OpenAI data format, we prepended the following System Prompt to strictly define the model’s persona: *“You are an experienced psychological expert skilled in applying Helping Skills, including the three-stage method of Exploration, Comfort, and Action, to assist clients in coping with emotional distress. In multi-turn interactions, you need to provide profound emotional support and practical advice through empathy, understanding, and guidance. You should flexibly adjust your responses based on the client’s feedback to ensure they align with the client’s context and needs. Through meticulous questioning and understanding, help the client deeply explore their feelings and problems, and ultimately guide them to find solutions. Please avoid dogmatic responses; instead, bring practical help and encouragement by respecting the client’s feelings.”*

### 5.3 Experimental setup

Model training was conducted on an NVIDIA A800 (64GB) GPU. During the training process, gradient accumulation steps were set to 8, accumulating gradients over 8 steps before each optimizer update. The learning rate was initialized at 3 × 10^−5^, employing a cosine learning rate scheduler for dynamic adjustment throughout the 3-epoch training duration. To accelerate training and optimize computational efficiency, we utilized FP16 mixed-precision training. Notably, the fine-tuning framework was implemented using LLaMA Factory, an efficient toolkit for large model adaptation.

### 5.4 Automatic evaluation metrics

To provide a multi-dimensional assessment of the model’s performance in psychological support dialogue tasks, this study adopted three categories of mainstream evaluation metrics:

- *N-gram overlap and lexical similarity:* we employed BLEU-1/2/3 and ROUGE-L to measure the precision of exact matches and the recall of the longest common subsequence between generated responses and reference texts, respectively.
- *Semantic relevance:* we introduced METEOR and BERTScore. METEOR incorporates synonym and stem matching, while BERTScore calculates cosine similarity in the embedding space to evaluate deeper semantic alignment.
- *Generation diversity:* distinct-1/2/3 were used to quantify the richness and non-repetitiveness of the generated content.

Regarding computational settings, all metrics (excluding BERTScore) were calculated based on Chinese character-level tokenization to mitigate biases introduced by word segmentation errors. For BERTScore, we selected the BAAI/bge-m3 model (Chen J. et al., 2024) as the underlying encoder to ensure precise semantic alignment in Chinese psychological support dialogues.

### 5.5 LLM-based automatic evaluation and pairwise comparison

To evaluate the practical impact of distinct data construction paradigms on downstream dialogue model performance—and specifically to assess generalization capabilities across diverse data sources—we conducted a rigorous pairwise comparison protocol.

#### 5.5.1 Experimental subjects and settings

We first define the comparative models and experimental conditions:

1. *HCoT-chat (ours):* a psychological support dialogue model developed by fine-tuning the base model Qwen2.5-7B-Instruct via LoRA, utilizing the HCoT-Corpus constructed in this study.
2. *MeChat (baseline):* to benchmark our method, we selected MeChat (Qiu et al., 2024) as a robust baseline. This system is a psychological support dialogue model fine-tuned on the SMILECHAT dataset, which was generated by rewriting the PsyQA dataset using the SMILE method.

By comparing HCoT-Chat with MeChat, we aim to determine whether HCoT-Chat outperforms the baseline MeChat under differing data construction strategies.

#### 5.5.2 Evaluation procedure

We employed a pairwise comparison protocol. We randomly extracted 100 multi-turn dialogues each from the HCoT-Corpus test set (in-domain distribution) and the SMILECHAT test set (out-of-domain distribution) to serve as the foundational evaluation sets. For each sample, both HCoT-Chat and MeChat generated responses based on the same dialogue history and Seeker inquiry.

To ensure robustness and mitigate single-viewpoint bias, we retained the multi-model independent arbitration mechanism validated in Section 4.4.4. We utilized a cross-architectural panel comprising GPT-5.2, Gemini-3, and Grok-4.1 as third-party arbiter models.

During the evaluation, each arbiter independently scored responses across the seven dimensions of the Bench framework (Section 4.4.1) and rendered a Final Decision. To enhance stability and reproducibility, the temperature parameter was uniformly set to 0.3, with default settings applied elsewhere. Instances where model performance was deemed indistinguishable were classified as a Tie and excluded from the win counts of either side.

#### 5.5.3 Result analysis

**Figure 8:**
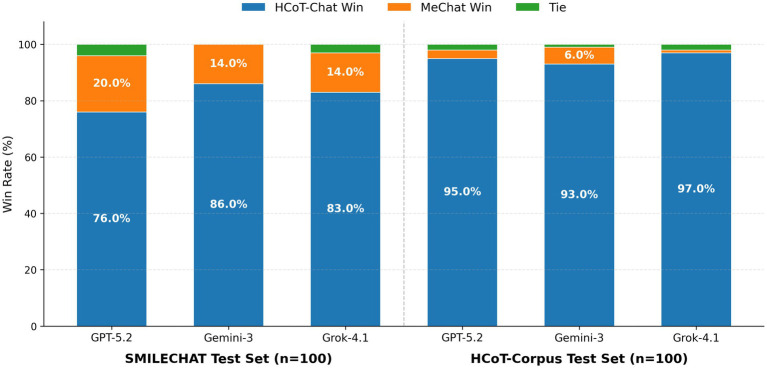
Pairwise win rate evaluation based on three major models. The left panel shows the generalization performance of the model on the SMILECHAT test set, while the right panel shows the professional performance on the in-domain HCoT-Corpus test set. The blue areas represent the proportion of wins for HCoT-Chat. The results indicate that HCoT-Chat maintains a significant advantage across all evaluator perspectives, achieving a particularly high win rate in the in-domain test.

Experimental results are visualized in Figure 8. The findings reveal the following:

1. *Out-of-domain distribution test (SMILECHAT Test Set):* On the native distribution of the baseline MeChat, HCoT-Chat demonstrated remarkable generalization capabilities. Judgments from the three arbiter models were highly consistent, with HCoT-Chat’s win rate ranging from 76.0 to 86.0% (mean 81.7%), significantly outperforming the baseline MeChat (14.0 to 20.0%) (Tables 6, 7). This indicates that even on non-fine-tuned data sources, HCoT-Chat secures consistent preference from major evaluator models. This empirical evidence helps alleviate concerns that the model is merely engaging in “rote memorization” of the training data. For detailed multi-dimensional scoring of this test set under the three evaluators, please refer to Appendix B.
2. *In-domain distribution test (HCoT-Corpus Test Set):* On the data distribution constructed in this study, the performance gap widened further. Leveraging the alignment between training data and testing scenarios, HCoT-Chat’s win rate surged to the 93.0 to 97.0% range, whereas the baseline remained in the single digits. This confirms that the HCoT framework possesses clear procedural utility in constructing structured, theoretically grounded psychological support dialogues.
3. *Performance Attribution Analysis:* Synthesizing the dimensional scores (see Table 8), HCoT-Chat’s advantage primarily stems from the deep application of empathy strategies. While the baseline model occasionally scored higher in the *[Direct Guidance]* dimension (e.g., Grok-4.1 score of 7.18 vs. HCoT-Chat’s 6.33), HCoT-Chat achieved a substantial lead in emotional support dimensions such as *[Restatement]* and *[Self-disclosure]* (mean score > 2.0, vs. baseline < 0.6). This result suggests that state-of-the-art evaluator models prioritize responses exhibiting deep emotional connection over those simply providing instructional advice when determining quality.

**Table 6:** Hyperparameters for parameter-efficient fine-tuning.

Epoch	Learning rate	Batch size	LoRA rank	LoRA dropout	LoRA α	Seed
3	3e-5	8	8	0.1	16	42

**Table 7:** Automatic evaluation results on the test set.

Models	METEOR	B-1	B-2	B-3	R-L	D-1	D-2	D-3	BERTScore
Qwen2.5-7B-Instruct	28.48	12.43	7.22	4.37	12.79	53.19	84.46	93.91	74.19
HCoT-Chat	33.17	37.43	24.28	17.11	30.66	85.69	98.17	99.66	78.59

**Table 8:** Multi-dimensional scoring comparison based on three large models on the HCoT-Corpus test set.

Evaluation dimensions	GPT-5.2		Gemini-3		Grok-4.1	
	HCoT	MeChat	HCoT	MeChat	HCoT	MeChat
Information	3.93	3.40	5.53	4.74	5.21	4.06
Direct Guidance	4.84	5.32	7.01	6.71	6.33	7.18
Approval and Reassurance	6.44	6.15	8.49	7.02	8.27	7.59
Restatement	5.60	2.72	8.22	4.32	7.89	3.10
Interpretation	5.59	3.14	7.78	4.12	6.20	3.15
Self-disclosure	3.02	0.13	4.96	0.55	2.52	0.35
Gathering	0.82	1.64	2.14	3.59	1.49	2.63
Total Score	30.30	22.54	44.13	31.05	37.91	28.19

In summary, HCoT-Chat demonstrated consistent and significant superiority across both in-domain and out-of-domain test sets. This validates the model’s robustness when facing diverse dialogue sources and its alignment with the empathy-centric counseling paradigm advocated by Helping Skills Theory.

## 6 Conclusion

This paper proposes the HCoT method, which integrates Helping Skills Theory with the Chain-of-Thought mechanism, and constructs the Chinese multi-turn psychological support corpus, HCoT-Corpus. This work achieves the structured generation of multi-turn strategic dialogues from single-turn Q&A pairs. Systematic analysis demonstrates that the corpus exhibits high structural consistency and multi-strategy collaborative characteristics under the “Exploration—Comfort—Action” framework. Subsequent comparative experiments further confirm that the HCoT method significantly outperforms baselines such as SMILE in both strategy adherence and dialogue quality. Model evaluation reveals that HCoT-Chat, fine-tuned on this corpus, not only surpasses the Qwen baseline across automatic metrics like METEOR and BERTScore but also achieves a consistent and significant advantage over MeChat in cross-architecture multi-model evaluations on both in-domain and out-of-domain test sets.

In summary, as a methodological proof-of-concept, this study confirms the preliminary feasibility of the HCoT framework, establishing it as a promising pathway for constructing large-scale, theoretically grounded datasets.

Furthermore, this study emphasizes that the procedural success of the generation tool is only a “necessary but not sufficient condition” for building high-quality datasets. Given the idealized characteristics of current synthetic data, HCoT-Corpus is currently positioned as an empirical basis for verifying the potential of the generation method. To support clinical application, future work must introduce licensed therapists to conduct “high-burden” strict validation, focusing on evaluating the realism of dialogues (especially the simulation of real resistance and non-linear dynamics) and clinical quality. Only after such dual validation at the clinical level can datasets based on this method be recommended for developing mental health applications for real users.

### 6.1 Limitations

Although the HCoT-Corpus constructed in this paper demonstrates innovation in theoretical framework and data generation, as a methodological proof-of-concept, this study remains subject to the following limitations:

First, there is room for optimization in the generation quality. HCoT-Corpus was rewritten based on GPT-4o; while it embodies the structured features of Helping Skills, compared to real counseling, it still exhibits issues of stylistic standardization and insufficient diversity. The use of strategies in the “Comfort Stage” is relatively weak in some dialogues, and transitions between stages are occasionally unnatural, suggesting that the model requires further improvement in strategy pacing control.

Secondly, synthetic data exhibits an “Idealized Collaborative Bias.” Since both roles (Seeker and Supporter) in HCoT-Corpus are simulated by LLMs, the dataset presents an “idealized hyper-cooperative interaction.” Compared to real clinical transcripts, this dataset lacks the psychological resistance, non-compliance, and linguistic ambiguity common in real clients. The model learns a standardized counseling path; therefore, performance degradation may occur when facing real users with high defense mechanisms or crisis intervention needs. Future research requires the introduction of de-identified clinical data and samples generated by experts containing “critical events” and “real resistance” to bridge the “simulation-reality gap.”

Finally, the evaluation paradigm needs to evolve toward industrial standards for larger-scale, higher-burden data construction. Given that this study is positioned as a preliminary methodological verification, we adopted a low-burden strategy of “trained rater verification + automated cross-model cross-validation.” While sufficient to support current conclusions, it is necessary to establish a “Human–AI Collaborative Ensemble Evaluation Framework” for future large-scale data construction. Future work will shift from “consistency checks” to “score fusion,” integrating weighted scores from heterogeneous LLMs and introducing Intraclass Correlation Coefficient (ICC) calibration to reduce single-model variance and establish more rigorous automated evaluation standards.

### 6.2 Suggested research applications and responsible use

Despite being positioned as a methodological proof-of-concept, HCoT-Corpus remains an important empirical resource. Based on principles of responsible use, we suggest the following research directions:

1. Simulated Counseling Environment Testing: Conducting “Human-in-the-Loop” simulated interactions involving trained actors to safely assess model risk boundaries and strategy effectiveness in non-clinical environments, accumulating empirical data for future higher-burden clinical expert validation.
2. Comparative Studies with Real Corpora: Quantitatively comparing differences in linguistic style and dynamics between synthetic data and real counseling records, identifying the gap between “idealized models” and “clinical reality,” and guiding algorithm optimization.
3. Development of Expert Evaluation Protocols: Using the detailed annotations of the dataset as “anchor data” to calibrate human expert scoring standards or train medical students to identify helping strategies, filling the gap in unified evaluation standards.

In summary, HCoT-Corpus aims to build a bridge connecting “AI Technology Construction” and “Clinical Validation,” and we encourage the community to promote the standardized development of mental health LLMs under this framework.

### 6.3 Ethical statement

The CD-CN dataset used in this study originates from the “YiXinLi” public platform and has undergone strict de-identification processing by the provider. Given that HCoT-Corpus is positioned as a “Methodological Proof-of-Concept” and has not been validated by clinical experts, it is strictly prohibited to directly deploy it or related models in clinical counseling services for real users. Responsible use of this data is limited to academic research (e.g., exploration of generation mechanisms, analysis of strategy patterns, and experimental model training). To clarify boundaries, the following scenarios are defined as examples of “Irresponsible Use”:

1. *Direct crisis intervention:* Using the model for suicide intervention or severe trauma treatment without integrating expert-level “critical event” modules;
2. *High resistance assumption:* Erroneously assuming the model can handle aggressive language or extreme non-compliance (due to the lack of such samples in the data);
3. *Unsupervised deployment:* Allowing the model to conduct unstructured, long-term counseling without human supervision.

Any actual intervention attempt without introducing human expert supervision is considered irresponsible use and may trigger safety risks. Therefore, this dataset serves solely as a methodological research resource rather than a clinical tool, and any experimental application must undergo strict manual screening to ensure ethical compliance.

## Data Availability

The datasets presented in this study can be found in online repositories. The names of the repository/repositories and accession number(s) can be found at: https://modelscope.cn/models/dlq1998/HCoT-Corpus.
