# Supplementary material for: Constructing and applying a multi-turn psychological support dialogue corpus based on the Helping Skills Chain-of-Thought
Source: Front Psychol. 2026 Mar 16;17:1733384. doi: 10.3389/fpsyg.2026.1733384 (PMC13033663; doi:10.3389/fpsyg.2026.1733384)
Supplement: Supplementary file 1 [file Data_sheet_1.pdf]

Appendix A. Data Preprocessing Pipeline

A.1 Automated Cleaning

We design and implement a sequential substitution pipeline to eliminate or replace forum-style expressions in the corpus, preventing potential interference with dialogue modeling. Specifically, expressions such as "楼主你" (literally thread starter you), "题主你" (literally poster you), and "楼楼你" (a colloquial term for thread starter you) are uniformly replaced with "你" (you). Similarly, terms like "楼主" (thread starter), "题主" (poster), and "楼楼" (colloquial thread starter) are replaced with "你" (you). Furthermore, semantically irrelevant colloquialisms, such as "嗨" (Hi) and "如题" (as titled), are removed. For specific nicknames (e.g., "阿琪"), we substitute them with the first-person pronoun "我" (I), while "答主" (respondent) is replaced with "人" (person). It is necessary to perform this replacement in a strict sequential order to avoid the repetition of "你" (you) and the resulting redundant "你你" (you-you).

A.2 Manual Cleaning

Due to the specificity and complexity of natural language, manual cleaning remains an essential part of the process. Following the automated cleaning, we conduct spot checks and revisions on the samples to manually delete redundant expressions that are difficult for the automated pipeline to identify. We also remove internet-slang expressions inappropriate for professional psychological counseling scenarios; for instance, we identify instances of the Chinese word for "hug" (抱抱) and manually delete or modify sentence snippets containing this term to prevent overly affectionate behavior.

Table A.1 illustrates a selection of the word substitution rules utilized during data cleaning. This table is adapted from the cleaning workflow in **SMILE (Sun et al., 2023)** and adjusted according to the specific corpus characteristics of this study.

Table A.1 Example of Word Substitution Rules in Data Cleaning

Old String (ZH)	Old String (EN)	New String (ZH)	New String (EN)
'嗨，'	Hi,	''	/
'抱抱'	hug	''	/
'给你一个温暖的抱抱'	give you a warm hug	''	/
'楼主你'	thread starter you	'你'	you
'题主你'	thread starter you	'你'	you
'楼主'	thread starter	'你'	you
'题主'	thread starter	'你'	you
'阿琪'	A Qi (a name)	'我'	me
'哭了'	cried / crying	'我很难过'	I feel sad

Appendix B. Detailed Evaluation Data for Cross-Architecture Multi-Model Arbitration

This appendix provides the detailed scoring data generated by the third-party arbiter array, comprising **Gemini-3** and **Grok-4.1**. These data serve to supplement the primary evaluation results based on GPT-5.2 presented in the main text and to further validate the **robustness** of our findings.

B.1 Cross-Model Evaluation Results for Data Generation Methods (Corresponding to Section 4.4.4)

This section presents the detailed dimensional scores and optimal preference distributions for the three generation methods (**HCoT**, **3Stage**, and **SMILE**) across different evaluators.

Table B.1: Grok-4.1 Automatic Scoring Results of Three Methods Across Seven Psychological Support Dimensions.

	SMILE	3Stage	HCoT
Information	5.20	5.41	6.79
Direct Guidance	6.72	7.34	8.19
Approval and Reassurance	6.96	8.11	8.25
Restatement	5.92	7.29	7.72
Interpretation	5.26	6.13	7.23
Self-disclosure	0.25	0.23	5.89
Gathering	7.63	8.73	4.35
Total	37.94	43.24	48.42

Table B.1: Gemini-3-Pro-Preview Automatic Scoring Results of Three Methods Across Seven Psychological Support Dimensions.

	SMILE	3Stage	HCoT
Information	5.62	6.13	7.14
Direct Guidance	6.36	7.23	7.61
Approval and Reassurance	6.86	8.22	7.71
Restatement	6.32	7.44	7.49

Interpretation	5.84	7.09	7.61
Self-disclosure	0.42	0.50	7.34
Gathering	6.87	8.26	4.72
Total	38.29	44.87	49.62

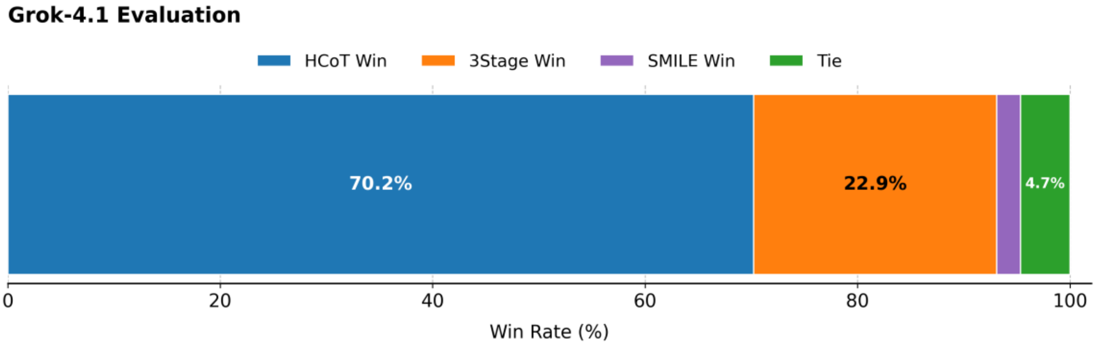

Figure B.1: Distribution of optimal preferences for the three methods based on Grok-4.1.

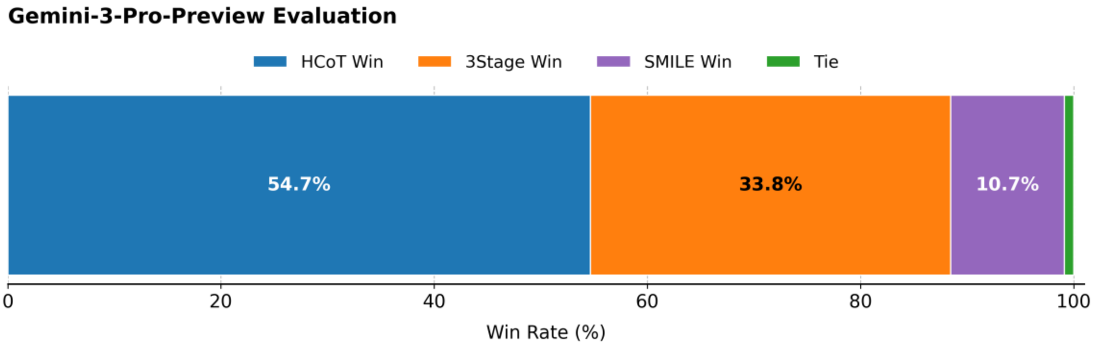

Figure B.2: Distribution of optimal preferences for the three methods based on Gemini-3-Pro-Preview.

B.2 Multi-dimensional Scoring Details for Dialogue Model Performance Comparison

This section presents the detailed performance comparison between HCoT-Chat and the baseline model MeChat on the out-of-domain test set (SMILECHAT). Table B.3 summarizes the independent scores assigned by three evaluators with different architectures (GPT-5.2, Gemini-3, and Grok-4.1) across seven dimensions for both models.

Table B.3. Multi-dimensional scoring comparison based on three large models on the SMILECHAT test set.

Evaluation Dimensions	GPT-5.2		Gemini-3		Grok-4.1	
	HCoT	MeChat	HCoT	MeChat	HCoT	MeChat
Information	2.98	3.53	3.86	4.24	3.69	3.76
Direct Guidance	4.01	5.61	5.41	6.97	4.16	6.79
Approval & Reassurance	6.65	5.89	8.86	6.94	7.78	6.34
Restatement	6.79	2.60	8.23	4.23	7.29	2.82
Interpretation	5.27	3.24	7.12	4.09	5.24	3.01
Self-disclosure	2.20	0.03	3.54	0.12	1.43	0.03
Gathering	1.14	4.14	3.00	5.62	2.73	5.31
Total Score	29.10	25.04	40.02	32.21	32.32	28.31

Appendix C. Qualitative Case Analysis

Figure C.1: A dialogue example generated with the HCoT method.

Dialogue in Chinese	Dialogue in English
求助者：为什么总会想着讨好别人？其实内心非常不愿意。	Seeker: Why do I always feel compelled to please others, even though deep down I really don't want to?
支持者[Restatement]：你感到困惑，因为你经常想要去讨好别人，而内心却对此感到抗拒。	Supporter [Restatement]: You feel confused because you often try to please others, yet internally you resist doing so.
求助者：是的，我感觉每次都很难受，但有时候又觉得必须得去讨好。	Seeker: Yes. It feels awful every time, but sometimes I also feel like I have to do it.
支持者[Interpretation]：听起来，你可能在内心深处感受到压力，可能是因为你想要获得某种认可或避免冲突。	Supporter [Interpretation]: It sounds like there may be pressure deep down—perhaps because you want to gain approval or avoid conflict.
求助者：可能吧，我总觉得如果不讨好别人，就会有麻烦。	Seeker: Maybe. I always feel that if I don't please people, there will be trouble.
支持者[Information]：很多时候，讨好行为可能源于对安全感的需求或害怕被拒绝，这在心理学上被称为讨好型人格。	Supporter [Information]: Often, people-pleasing can come from a need for security or a fear of rejection. In psychology, this is sometimes described as a "people-pleasing personality."
求助者：原来如此，我确实经常害怕别人不喜欢我。	Seeker: I see. I really do often fear that others won't like me.
支持者[Self-disclosure]：我曾经也有过类似的感觉，害怕别人不认可自己，希望能通过一些努力来获得他们的喜欢。	Supporter [Self-disclosure]: I've had similar feelings before—fearing that others wouldn't approve of me and hoping I could earn their liking by trying harder.
求助者：那你是怎么处理这种感觉的？	Seeker: So how did you deal with that feeling?
支持者[Direct Guidance]：重要的是认识到你自己的价值，而不是通过别人的认可来定义自己。你已经很努力了，值得被理解和支	Supporter [Direct Guidance]: What matters is recognizing your own worth, rather than defining yourself through other people's approval. You've already been trying hard, and you

持。	deserve understanding and support.
求助者：谢谢你，我确实需要更多地关注自己。	Seeker: Thank you. I really do need to pay more attention to myself.
支持者[Approval and Reassurance]：关注自己的感受是非常重要的一步，你正在往正确的方向迈进。	Supporter [Approval and Reassurance]: Paying attention to your feelings is a very important step—and you're moving in the right direction.
求助者：我想知道怎么才能不总是想着去讨好别人。	Seeker: I want to know how I can stop constantly trying to please others.
支持者[Direct Guidance]：你可以开始尝试设定个人界限，并明确自己的原则，这样在面对需要讨好他人的情况时，能更好地坚持自己的立场。	Supporter [Direct Guidance]: You can start by trying to set personal boundaries and clarifying your principles, so that when you feel pressured to please others, you can better hold your ground.
求助者：设定界限听起来不错，但我总是担心会得罪人。	Seeker: Setting boundaries sounds good, but I'm always worried about offending people.
支持者[Interpretation]：设定界限并不意味着会得罪人，而是表达你自己的需求和限制。许多人会尊重这种坦诚。	Supporter [Interpretation]: Setting boundaries doesn't necessarily mean offending others—it means expressing your needs and limits. Many people will respect that kind of honesty.
求助者：我会尝试这样做，希望自己能够慢慢改变。	Seeker: I'll try that. I hope I can change gradually.
支持者[Approval and Reassurance]：这是一个积极的开始，改变需要时间，你已经在努力，并且值得为自己感到骄傲。	Supporter [Approval and Reassurance]: That's a positive start. Change takes time—you're already making an effort, and you have reason to feel proud of yourself.
求助者：谢谢你的支持，我会继续努力的。	Seeker: Thank you for your support. I'll keep working on it.
支持者[Approval and Reassurance]：我相信你可以做到，勇敢做自己，你会发现更多的幸福和快乐。	Supporter [Approval and Reassurance]: I believe you can do it. Be brave in being yourself, and you'll find more happiness and joy.

Figure C.2: A dialogue example generated with the SMILE method.

Dialogue in Chinese	Dialogue in English
求助者：我总是觉得自己在讨好别人，内心其实很不愿意这样做。	Seeker: I always feel like I'm pleasing others, but deep down I'm really unwilling to do so.
支持者：你好，听起来你对这种情况感到很困扰。能告诉我更多关于你为什么觉得需要讨好吗？	Supporter：Hello, it sounds like you are troubled by this situation. Can you tell me more about why you feel the need to please others?
求助者：可能是因为我想要获得别人的认可和赞美。	Seeker: It might be because I want to gain others' approval and praise.
支持者：了解了，许多人会因为想得到认可而去讨好别人。你觉得这对你来说是一种习惯吗？	Supporter: Understood. Many people please others because they want to gain approval. Do you feel this is a habit for you?
求助者：是的，我总是控制不住自己去迎合别人。	Seeker: Yes, I can't control myself from catering to others.
支持者：这可能是因为你对别人的感受特别敏感。你有没有注意到自己在这些情况下会特别不安？	Supporter: This might be because you are particularly sensitive to others' feelings. Have you noticed if you feel particularly uneasy in these situations?
求助者：确实，我总是担心别人对我的看法。	Seeker: Indeed, I am always worried about others' opinions of me.
支持者：这种担心可能使你更愿意迎合他人。尝试给自己一些空间，去感受自己的需求和愿望。	Supporter: This worry might make you more willing to cater to others. Try giving yourself some space to feel your own needs and desires.
求助者：但是，我很难拒绝别人，总觉得要让大家都满意。	Seeker: But, it's hard for me to refuse others; I always feel I need to satisfy everyone.
支持者：拒绝别人确实不容易，但设定个人界限是重要的。你有没有试过在小事情上先表达自己的真实想法？	Supporter: Refusing others is indeed not easy, but setting personal boundaries is important. Have you tried expressing your true thoughts on small matters first?
求助者：没有，我总害怕这样做会让别人不高兴。	Seeker: No, I'm always afraid that doing so will make others unhappy.
支持者：这种害怕是可以理解的，但也许你	Supporter: This fear is understandable, but perhaps you can

可以从小事开始练习，慢慢建立自己的原则。	start practicing with small things and slowly build your own principles.
求助者：我怕这样会被误解为自私。	Seeker: I'm afraid this will be misunderstood as selfishness.
支持者：自私和自我关爱是不同的。学会爱自己并不是忽视他人，而是关注自己的感受和需求。	Supporter: Selfishness and self-care are different. Learning to love yourself is not ignoring others, but focusing on your own feelings and needs.
求助者：那我该如何开始爱自己呢？	Seeker: So how should I start loving myself?
支持者：可以尝试做一些让自己感到快乐和有价值的事情，慢慢地，你会发现自己不需要通过讨好来获得认可。	Supporter: You can try doing things that make you feel happy and valuable. Slowly, you will discover that you don't need to gain approval through pleasing others.
求助者：谢谢，我会尝试关注自己的感受和需求，希望能有所改变。	Seeker: Thank you, I will try to focus on my feelings and needs, hoping for some change.
支持者：很高兴你愿意尝试。记得给自己时间和耐心，慢慢来。你值得拥有幸福和快乐。	Supporter: I'm glad you are willing to try. Remember to give yourself time and patience; take it slow. You deserve happiness and joy.

Appendix D. System Prompts for Automated Evaluation

Table D.1: The System Prompt for LLM-based Automatic Evaluation.

[System Instruction] You are a neutral and objective psychological dialogue arbiter. You will be provided with three multi-turn psychological counseling dialogues (A/B/C) generated by different models based on the same user query.<br>Task: Compare the pros and cons of the three dialogues in terms of response quality, structural integrity, and strategic application, strictly based on the defined counseling strategies below.
**[Definitions of Helping Skills Strategies]** Please score each dialogue (0-10) based on the following seven dimensions:<br>1. **Information**: Providing data, facts, opinions, or resources relevant to the user's issue.<br>2. **Direct Guidance**: Offering advice, instructions, or suggestions on how the seeker should change or act.<br>3. **Approval & Reassurance**: Providing emotional support, encouragement, and reinforcement to enhance the seeker's sense of security and confidence.<br>4. **Restatement**: Repeating or rephrasing the content or meaning of the seeker's statement more clearly and concretely.<br>5. **Interpretation**: Going beyond what the seeker has explicitly stated to provide new meanings, causes, or explanations.<br>6. **Self-disclosure**: Revealing the supporter's own personal experiences or feelings (non-immediate) to facilitate empathy.<br>7. **Gathering**: Asking appropriate questions to collect necessary details for assessment.
**[Evaluation Constraints]**

- **No Bias:** Do not favor a dialogue based on its order (e.g., A first).
- **Focus:** Focus on the **quality of strategy use** and the **completeness of the support structure**, not just length or tone.
- **Differentiation:** Avoid giving identical scores; try to distinguish the performance of each strategy.

[Output Format] Please output **ONLY** a JSON object without any markdown or explanations:

```
{
 "scores": {
  "A": {
   "information": 8, "direct_guidance": 9, "approval_reassurance": 7,
   "restatement": 6, "interpretation": 8, "self_disclosure": 5,
   "gathering": 7, "total": 50
  },
  "B": { ... },
  "C": { ... }
 },
 "final_decision": "A/B/C/TIE",
 "explanation": "Briefly explain the reason, highlighting strategy use and structure."
}
```
